# Utility of the three-delays model and its potential for supporting a solution-based approach to accessing intrapartum care in low- and middle-income countries. A qualitative evidence synthesis

**DOI:** 10.1080/16549716.2020.1819052

**Published:** 2020-10-12

**Authors:** Valentina Actis Danna, Carol Bedwell, Sabina Wakasiaka, Tina Lavender

**Affiliations:** a Department of International Public Health, Liverpool School of Tropical Medicine, Liverpool, UK; b College of Health Science, School of Nursing, University of Nairobi, Nairobi, Kenya

**Keywords:** Maternal care, health empowerment, individualised-care, three delays model, qualitative evidence synthesis, childbirth, obstetric care

## Abstract

**Background:**

The 3-Delays Model has helped in the identification of access barriers to obstetric care in low and middle-income countries by highlighting the responsibilities at household, community and health system levels. Critiques of the Model include its one-dimensionality and its limited utility in triggering preventative interventions. Such limitations have prompted a review of the evidence to establish the usefulness of the Model in optimising timely access to intrapartum care.

**Objective:**

To determine the current utility of the 3-Delays Model and its potential for supporting a solution-based approach to accessing intrapartum care.

**Methods:**

We conducted a qualitative evidence synthesis across several databases and included qualitative findings from stand-alone studies, mixed-methods research and literature reviews using the Model to present their findings. Papers published between 1994 and 2019 were included with no language restrictions. Twenty-seven studies were quality appraised. Qualitative accounts were analysed using the ‘best-fit framework approach’.

**Results:**

This synthesis included twenty-five studies conducted in Africa, Asia, Latin America and the Caribbean. Five studies adhered to the original 3-Delays Model’s structure by identifying the same factors responsible for the delays. The remaining studies proposed modifications to the Model including alterations of the delay’s definition, adding of new factors explaining the delays, and inclusion of a fourth delay. Only two studies reported women’s individual contributions to the delays. All studies applied the Model retrospectively, thus adopting a problem-identification approach.

**Conclusion:**

This synthesis unveils the need for an individual perspective, for prospective identification of potential issues. This has resulted in the development of a new framework, the Women’s Health Empowerment Model, incorporating the 3 delays. As a basis for discussion at every pregnancy, this framework promotes a solution-based approach to childbirth, which could prevent delays and support women’s empowerment during pregnancy and childbirth.

## Background

Around 295,000 maternal deaths occurred in 2017, with the highest toll paid by Sub-Saharan Africa and South Asia [1]. The global Maternal Mortality Ratio has declined by 38% worldwide between 2000 and 2017, although disparities remain across regions with 415 maternal deaths per 100,000 live birth in low-income countries compared to 7–10 maternal deaths for 100,000 live birth in Europe, Australia and New Zealand [2].

Maternal death is often caused by obstetric complications arising during pregnancy and childbirth. However it is also influenced by indirect causes such as anaemia, malaria and heart diseases [3]. Most maternal deaths are preventable with timely access to intrapartum care [1].

In 1994, Thaddeus and Maine [4] proposed the Three Delays Model (3DM) to facilitate the identification of indirect factors that, from the onset of obstetric complications to the birth of the baby, contribute to maternal death. The Model identifies three critical phases which can have direct consequences on the survival of the mother and baby: delay in the decision to seek care (First Delay), delay in identifying and reaching the health facility (Second Delay), and delay in receiving appropriate treatment at the facility (Third Delay). The First Delay has been associated with family and community-related factors, such as the socio-economic status of the woman, knowledge of pregnancy danger signs and perceived severity of illness during pregnancy, perception of the physical distance to the health facility, potential cost of care and previous experience with the health system. The Second Delay refers to accessibility challenges, due to distance, availability and effective costs of means of transport; and the distribution of the health facilities in the area where the woman lives. The Third Delay is concerned with the service offered at the facility. This can be insufficient due to lack of supplies and equipment, unfriendly environment (including disrespectful care) and inadequate and poorly trained staff [5,6].

The Model adopts a holistic approach to understand the different responsibilities at household, community and health system levels to prevent maternal death. Its structure has made it a practical tool for the identification of context-specific challenges, targeting both users and providers [7–9]. Moreover, the Model has facilitated research into aspects of maternal health care in low and middle-income countries (LMICs), including maternal healthcare-seeking behaviours [10], the rationale for babies being born before arrival at the facility [11], and women’s preferences for home births [12].

Conversely, the Model has been critiqued for being too simplistic [13], one-dimensional [14] and sequential [6], and for lacking the complexity of more sophisticated models [15]. The framework is based on the assumption that women will only face delays when complications occur; whereas women often face delays without life-threatening conditions [15]. The original version gives limited attention to accessing preventive and postnatal care [15–17] and its application has not encouraged an action-oriented approach [14]. The Model has been used retrospectively to identify access barriers to maternal care [18]. This application has often led to formulating solutions to these barriers, rather than focusing on preventive interventions (e.g. a surveillance system to detect factors preventing adverse outcomes) [17]. Moreover, the Model does not capture the interplay between social and medical factors and their relationship with women’s individual needs. In many LMICs women’s voices on childbirth matters are still neglected when it comes to decision-making processes and actions related to their health [19,20]; and this is despite substantial progress in the reduction of global maternal mortality [2].

By reviewing studies which have applied the 3DM as a framework of analysis, this synthesis aims to determine if the Model is still appropriate in contemporary care and whether it can assist in the formulation of solutions which go beyond addressing the 3-delays barriers. We also assessed whether the Model could be reframed to integrate multi-sectoral, rights-based and gender-sensitive approaches promoting the empowerment of women as advocated in the Sustainable Development Goals [21].

## Methods

We conducted a qualitative evidence synthesis to analyse how the 3DM has guided authors’ analysis of participants’ experiences of accessing obstetric care in LMICs. Qualitative findings were chosen for the richness of in-depth experiences narrated by participants and to capture nuanced information from multiple perspectives. This facilitated the recognition of the existing Model’s categories, and the identification of new elements.

The focus on the Model’s use, led to the choice of the ‘best fit framework synthesis’ approach [22,23]. In this type of synthesis, primary studies are mapped against an *a priori framework*, to confirm existing data and to generate new interpretations [22]. The latter encompasses data not fitting in the a priori framework. Thereafter, a new framework is produced to integrate both existing and new evidence [22]. The Enhancing transparency in reporting the synthesis of qualitative research (ENTREQ) approach [24] was used to report findings of this exercise.

### Search strategy

Selected search terms were identified through an adapted version of the SPIDER tool for Qualitative Evidence Synthesis [25] (Table 1). The ‘design’ section was left open to avoid missing relevant papers.

**Table 1.** Search terms.

Sample	Woman OR mother OR pregnant OR parturient OR female
Phenomenon of Interest	Delays = delay OR wait OR time OR 3-delays AND Intrapartum care = Intrapartum OR delivery OR labour OR childbirth OR birth OR obstetric AND Low income settings = low income OR developing country OR LMIC OR LMICs OR sub-Saharan Africa OR Asia OR middle-income countries OR Latina America
Design	NA
Evaluation	Views OR opinions OR perceptions OR beliefs OR attitudes
Research type	Qualitative OR Mixed-method OR Phenomenology OR Grounded theory

The terms (Table 1) were used in different combinations to produce the highest number of results and were searched across several databases including MEDLINE, CINAHL Plus and Social Science Full text, Web of Science, Science Direct, Psych INFO, EMBASE, the Cochrane Library, the WHO Library for WHO databases, the African Journal Online, PROQUEST for dissertation and Thesis, Open Grey and Ethos for grey literature (Supplementary file). A number of papers and reports were also included by hand-checking the reference list of the included papers. An initial search was conducted by VAD in February 2017 with the support of the librarian, and was confirmed by CB. Iterative sampling continued until October 2019, to ensure the inclusion of new published literature. All authors agreed on the final eligibility of included studies based on inclusion and exclusion criteria.

### Inclusion and exclusion criteria

Studies were selected if they met the following criteria: 1) use of the 3DM as a guiding framework, including cases in which the Model had been modified; 2) use of the Model’s categories to present findings without an explicit mention in the methodology; 3) qualitative findings from mixed-methods and stand-alone qualitative research papers; 4) publication timeframe between 1994 (the 3DM’s year of publication) and 2019; and 5) studies published in any language. We excluded papers reporting only quantitative findings, not using the Model to assess access barriers to obstetric care, and not conducted in LMICs based on the World Bank classification [26].

### Quality appraisal

Hawker’s checklist [27] was used to assess the quality of included studies. This tool is appropriate when there are various paradigms and different research designs, as in this synthesis. The checklist was used to assess each study section, applying a four-point scale (‘Good = 4’, ‘Fair = 3’, ‘Poor = 2’, ‘Very Poor = 1’) system [27]. To ensure trustworthiness of the included evidence all the authors agreed that only studies with the highest grading (‘good’ and ‘fair’) would be included.

### Extraction and synthesis of data

Each paper was read thoroughly and classified according to the use of the 3DM. The first group included papers adopting the Model in their methodology; the second group contained studies in which the 3DM was not mentioned in their methods but was used to analyse findings; the third one involved papers proposing changes to the 3DM. Based on this classification, two matrixes were created to extract the following information from the included studies: country, study type, population and sample size, methods of data collection, factors contributing to each delay, changes proposed to the 3DM.

The 3DM was assessed in its entirety and for each delay to identify areas perhaps missed or over applied, and to eventually formulate a new interpretation of its components or of the full model. In synthesising qualitative research, the sum of how many times a phenomenon occurs (in this case the number of times each category of the 3DM was used) was not the main focus. Instead, we aimed to understand whether participants’ experiences were strictly mapped against the 3DM or if the Model needed an adaptation to capture emerging issues. Therefore, we compared each study with the categories of the 3DM and recorded in the matrixes how these were used to discuss access barriers to obstetric care. This entailed listing all factors falling under each category and highlighting new denominations or explanatory elements. The information derived from the first matrix was input into a spreadsheet organised around the First, Second and Third Delay’s. A compare and contrast exercise helped to determine how each study’s findings related to another with regard to the use of the Model’s categories. As a result of this, we highlighted the most used categories and confirmed new factors explaining the delays. The articles derived from the second matrix were also inputted into the spreadsheet to confirm the identified patterns and to draw attention to any alterations to the 3DM. The studies were then re-grouped according to changes proposed. An analysis of the questions posed to the participants (about barriers or solutions) helped to determine if the Model was used prospectively or retrospectively.

## Results

### Literature search and quality appraisal

The literature review produced 1,884 results, from which 617 duplicates were removed. Exclusions were mainly due to being irrelevant to the topic, published in high-income settings or before 1994. Fifty-nine articles were retrieved for full-text reading and 15 were added from hand-checking the reference lists of these papers.

**Figure 1.**
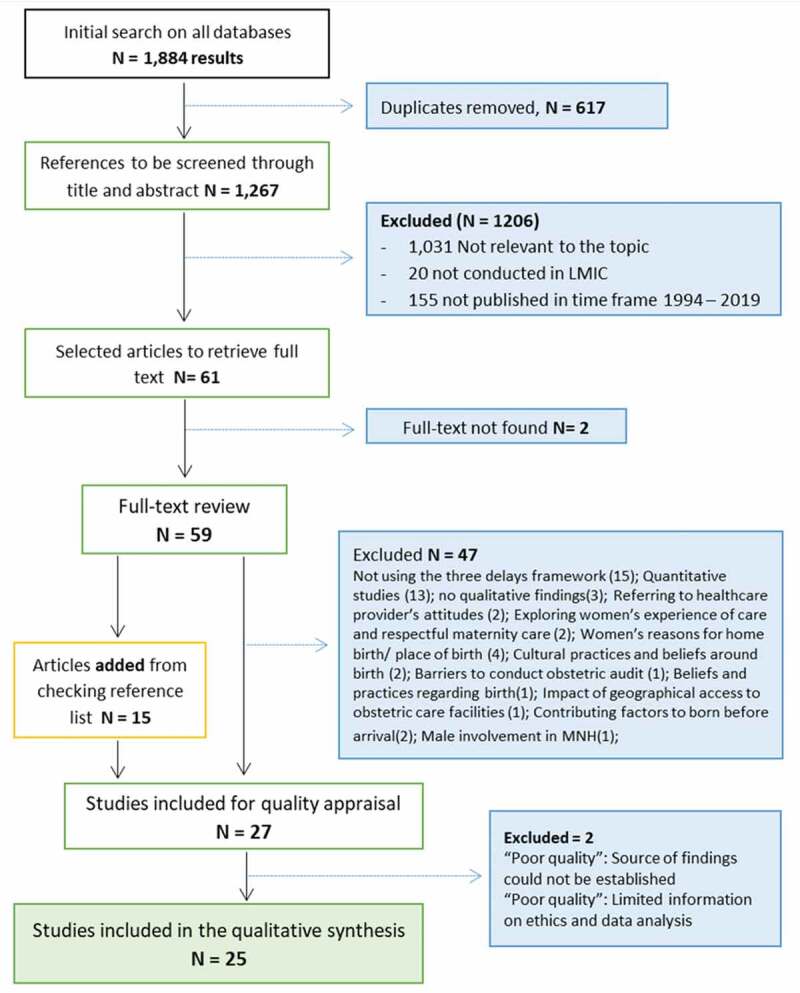
PRISMA flow chart.

From this review process (Figure 1), twenty-seven studies were included for quality appraisal. The latter established that eight studies were of good quality [9,14,15,28–32]; while 17 were of fair quality [6,7,33–47]. Two papers received a ‘poor’ quality grading [48,49] and were excluded. The final synthesis included 17 qualitative studies, 7 mixed-methods research papers and a literature review (Appendix A).

### General characteristics of included studies

The synthesis included twenty-five studies which were conducted in Democratic Republic of Congo [41], Ethiopia [32], The Gambia [33,40], Ghana [7], Kenya [43,46], Liberia [47], Malawi [15,34], Nigeria [9], Rwanda [31], Tanzania [14,44], India [35,37,42], Timor-Leste [30], Colombia [36], Haiti [29,45] and Mexico [39]. One of the multi-country papers involved Ethiopia, India, Indonesia, Nigeria, Tanzania, Uganda and Nepal [38]; the other one included Indonesia and Burkina Faso [6].

Eleven studies focused on maternal deaths [6,15,33–37,39,42,44,45]; 4 studies investigated both deceased mothers and: near-miss women [47], women who had post-partum haemorrhage [9,38], and women with obstetric complications [41]. Three papers concentrated on near-miss women [29,31,43]; the remaining studies involved women with various reproductive history [7,14,30,40,46]. The sample size for maternal death cases ranged from 10 to 403 women, for alive women involved between five and 208 individuals. Women’s age spanned from 12 to 49 years old, although in three papers it was not clearly indicated [7,33,38]. Data on maternal deaths were retrieved through verbal and social autopsies. The other studies collected information through in-depth interviews and focus groups discussions. Key informants included women, relatives, community members, traditional birth attendants and health workers. In the majority of papers, a thematic content approach was used to analyse findings. Five articles [36,37,39,42,44], reported few details about the indexing process.

Studies investigating maternal deaths asked participants to retrace the sequence of events leading to death, with a focus on barriers. Similarly, living women with different obstetric history [9,29–31,38,40,43,46] narrated difficulties of their last pregnancy after recovery. Experiences were all recounted retrospectively, thus an element of recall bias could be present and was acknowledged in some papers [6,9,15,38,40,41,47].

Women’s birth preparedness was explored in two studies [35,43], but not as a measure to prevent delays. In three articles [14,29,38] the 3DM was combined with another framework (the 5 C Model, the Pathways to Survival, the Actantial Model) to help in the identification of solutions to reduce maternal mortality.

### First delay – delay in the decision to seek care

In the 3-Delays Model (3DM), the factors influencing the First Delay were organised in three categories and related sub-categories.

#### Socio-economic and cultural factors

**Figure 2.**
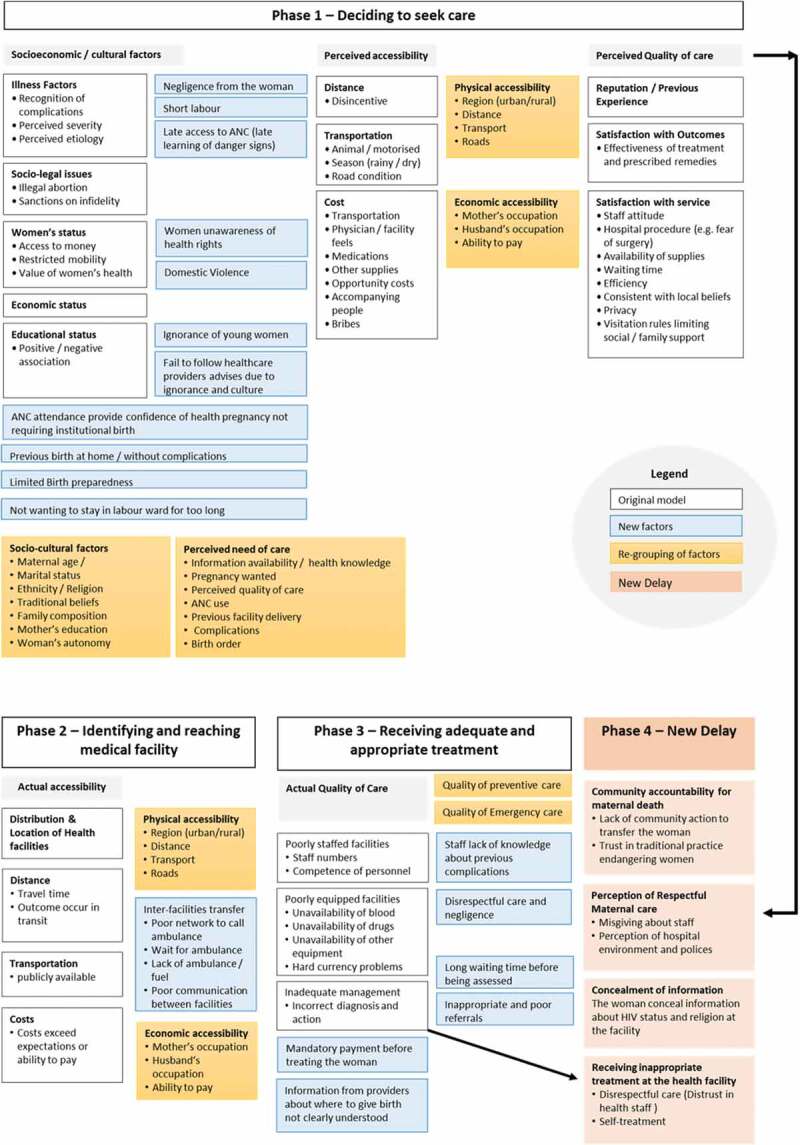
New contributing factors and re-grouping proposed to the three delays model.

The illness factor referred to the capacity of the woman to recognise the danger signs of pregnancy and judge the severity of her condition. In this synthesis, this category has remained important in understanding how women perceive the progress of their pregnancies and their actions when they suspect a problem or an increase in the severity of a condition. In the 3DM it was assumed that the woman has sole responsibility for these actions, however, included studies demonstrated more complexity. Findings indicate that knowledge of the danger signs is often limited [6,9,15,31,34–36,39]; when some women recognise the danger signs [32,38,40–43,46,47], they will either neglect them [29,39,41], or fail to perceive the severity of the complication to seek care on time [9,15,29,40,41]. In a few cases this unawareness was also dictated by a previous uneventful birth [35,46] taking place at home [33–35,37]; an aspect which was not previously acknowledged. In a number of studies, other new factors were added to explain this delay (Figure 2), including poor or late antenatal care attendance [35,36]; non-compliance with healthcare provider’s advice [14], aversion to prolonged labour ward stay [34], lack of birth preparedness [30,35], and domestic violence [37,39].

In the 3DM socio-legal issues, as sub-factor of socio-economic and cultural factors referred to illegal abortion and sanctions on infidelity as possible contributors to the First Delay. This synthesis found similar issues in India [42], Haiti [29] and Rwanda [31].

#### The status of the woman

Thaddeus and Maine [4] recognised that care-seeking decisions made by women are influenced by access to money and freedom of movement. This review illustrates that the decision to access care is often the prerogative of the husband [9,14,28,30,34,38–40,44,46] or of the mother-in-law [9,30,40,44,46] and, in their absence, of other family members [6,9,47]. In Haiti the absence of a male partner to go to the health facility [29] was also named among the reasons of the First delay. These findings highlight how the decision to seek care often seems to be largely determined by power relationships between the couple and the extended family, in addition to financial and mobility aspects.

#### Economic and educational status

The 3DM considered economic and educational status as contributing factors to the First Delay [4] but did not assess how these two variables influence the decision-making process. According to Thaddeus and Maine [4] a better economic status determined a higher utilisation of health services. This synthesis found that in several settings the lack of financial means [6,9,29–31,33,34,37,41,42,46] delayed families from the decision to seek formal care.

Educational status was included in the 3DM despite a limited evidence about how the woman’s level of schooling influenced healthcare-seeking decisions [4]. Three studies [7,29,35] referred to education among the reasons of the First delay; the remaining papers, included education-related details [6,15,29–31,34,36,37,40,41,45,47] to describe the characteristics of the sample population, but did not consider it as potential contributor for the First Delay.

#### Distance, transport and cost

Perceived accessibility to the health facility could influence the decision to seek care [4,28].

In the 3DM, the distance from home to the health facility plays a significant role in care-seeking decisions and longer distances can act as a disincentive, especially in rural areas. This is worsened by lack of transport and poor road conditions. Lastly, the indirect cost of seeking care given by transportation fees and hospital-related costs represents another deterrent. In this synthesis, few studies reported remoteness from health facilities [15,28,34,37] and availability of transportations [6,32] as reasons for the First Delay. In the majority of studies, delays in the decision to seek care due to perceived accessibility were driven by the potential cost of transport and for institutional care [9,28,29,31,35,39–41,45,46].

#### Quality of care

In the 3DM, the First delay could also be affected by previous experience with the health system [4,28]. Many studies in this synthesis have shown how a bad experience with health professionals [6,14,31,33–35,38,39,45], fear of medical procedures [37] and an unfriendly environment [9,30,33,46] could deter women from future appointments and delay their care-seeking decisions.

In this category, Thaddeus and Maine [4] recognised how beliefs and the use of traditional medicine could delay the decision to access care. The choice to consult traditional healers and use traditional birth attendants before seeking formal care was a recurrent situation in various countries [7,9,14,15,29,34,38,40,42,44,47]. This decision intended to comply with local beliefs and rituals [6,30–33,35,39], but was also implied by the possibility of delaying payments for care [31,46].

### Second delay – delay in identifying and reaching the health facility

The Second Delay was determined by the geographical distribution of facilities, distance from home to the facility, weak road infrastructure, availability of means of transports and costs [4]. These factors have been explored in the included studies. In some countries, living in remote and rural locations [15,32,37,40,47] characterised by poor road condition [7,32–35,37,43] delayed women from reaching care on time. Studies conducted in India, The Gambia and in Nairobi slums [37,40,46] showed how the rainy season transforms roads into muddy pathways, with impossible driveability. In the rural Gambia [40] living next to a river meant being subject to floods which affected the availability of ferry services to reach the mainland and access care. In a number of studies [14,29,33,39,44], long travel time due to distance was cited among the main challenges to reach healthcare promptly.

In the 3DM, availability and cost of public transports were also discussed [4]. In this review, lack of transportation was common across settings [6,7,9,14,15,35,38,39,42,45–47], especially in the absence of a motorised vehicle. Use of alternative means such as bicycles/motorbikes [6], animal carts [15,33,40,46,47] or, in extreme situation having to walk [33,46,47], did not address the accessibility need, as the journey was lengthened. Transportation issues were common at night due to service unavailability [15], unwillingness to travel on unsafe roads [47], increase in transports fares [46], and fear of thieves and wild animals [14,46]. It was noticed that transport arrangements were often made by relatives [9,14,15,30,32,33,35,39,40,44,45,47] and the community, if a local system was in place [6,30,33]. Only one article indicated that women organised their own transport [43].

In this synthesis, gaps in the referral system were discussed under the Second delay, an element not highlighted in the 3DM (Figure 2). The inability of initial facilities to provide basic or comprehensive emergency and obstetric care [35,37,39,46], meant that transfer was needed. Delays in this segment were due to lack of ambulances [30,33,40,43], fuel [33,46], and waiting for ambulance arrival [30]. These hindrances caused families to arrange their own transport to transfer the woman [14,35,46]. In one scenario, inter-facility referral lead to delay due to poor communication between and within facilities [31].

### Third delay – delay in receiving adequate and appropriate treatment at the facility

The Third Delay in the 3DM was influenced by a low number of staff, limited or reduced competences of providers, inadequate management, and shortage of equipment, medicines and blood [4]. In this synthesis these categories were still relevant in describing the challenges faced by many LMICs.

Across the studies, a limited number of human resources [6,7,31,35,36,39,44] and a lack of trained staff [15,34,35,38,44], especially doctors [33,37,43,44] delayed women from receiving appropriate care. This was compounded by the inability to diagnose obstetric complications [15,32,45] or for having made a wrong assessment [31,34,36,42,44], which in both cases could lead to inappropriate treatment [31,34,39,42,45].

Other sources of delays, not presented in the 3DM (Figure 2), included long waiting time before being assessed [6,34,43,46], inappropriate or poor referrals [7,14,33,37,38,43,46], and situations in which care was contingent to payments [6,41,46]. Thaddeus and Maine [4] acknowledged the impact of staff attitude on care-seeking decisions, but did not explore its contribution to the Third Delay. In this synthesis [29–32,39,41,46] negative attitudes, malpractice, limited interaction between women and the providers contributed to the Third Delay. As accounted in the 3DM, blood products [15,34–36,40,41,44], medicine and supplies [14,15,31–34,38,44,46] were insufficient in most settings, while equipment [6,7,34,37,39] including theatres [41,44] were sometimes unavailable.

### Changes proposed in the literature to the three delays model

**Figure 3.**
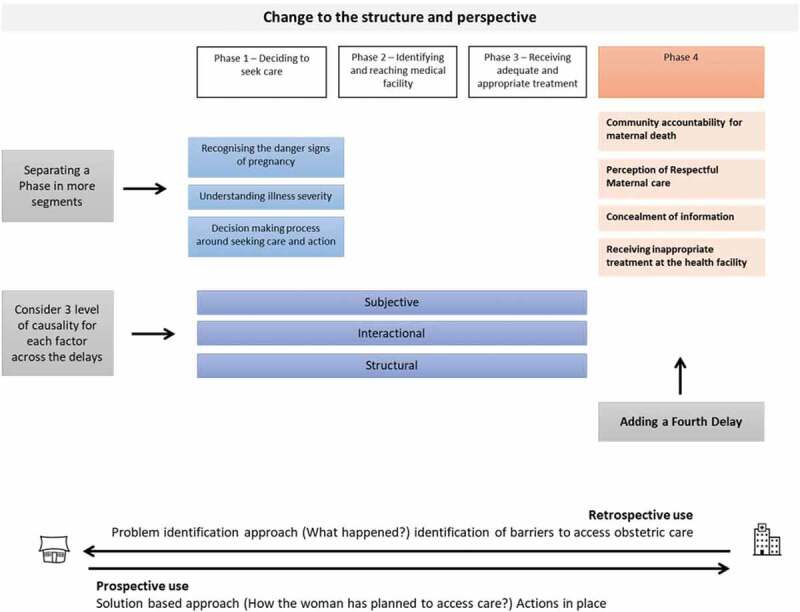
Changes proposed to the structure of the three delays model.

Ten of the 25 included studies proposed changes to the definition and structure of the 3DM (Figure 3).

#### Change in the definition of the delay

Three studies proposed changes in the definition of the First and Second Delays. Charlet et al. [38] proposed dividing the First Delay into three segments: the identification of life-threatening complications, the recognition of illness severity and the decision-making process around care-seeking to explore how the woman and her family interact in deciding where and when to seek care. Similarly, Rodriguez Villamizar et al. [36] separate the recognition of a problem from the decision to take action to identify the health needs and the factors influencing the decision to seek care. Jithesh and Ravindran [37] adapted the definition of Delay 1 and 2 to capture the time span taken to reach appropriate obstetric care due to multiple referrals.

#### The fourth delay

Four studies [15,29–31] included in this synthesis proposed a new delay to explain the journey to access care (Figures 2 and 3). Combs Thorsen et al. [15] identified the ‘Phase 3B Delay’ to indicate the delays due to women’s concealing information about their HIV status and religion, at the facility, which prevented staff providing timely care. For MacDonald et al. [29], a fourth delay explained the community role in contributing to maternal death. This involved a failed action to support women in reaching the health facility, and the pressure of the local culture to rely on traditional medicine instead of seeking institutional care as a first choice. Another suggestion has been separating the perception of respectful quality care from the factors determining the First Delay to recognise it as a unique delay [30]. Lastly Pafs et al. [31], added a new phase of delay to demonstrate how receiving inappropriate treatment compelled women to prolong their care-seeking journey.

#### Other changes to the three delays model

Another critique highlights the importance of separating the economic factors from the socioeconomic and cultural components to distinguish between economic and physical accessibility [28]. These re-groupings draw attention to physical access to care and perceived need of institutional care. Gabrysch and Campbell [28] suggest also a separate analysis of the determinants of care-seeking (decision-making, costs, distance, etc.) for preventive maternal care and emergency obstetric care. In their opinion, the different level of urgency of these situations influence the way the determinants interact and the possible occurrence of delays [28].

The 3DM has also been integrated with three levels of causality: structural, interactional and subjective, to understand maternal mortality in its complexity and to formulate solutions at each level [39]. Similarly, Sorensen and colleagues [14] suggest a need for a shift in the focus of the Model from determinants of care-seeking to provision of care closer to women, in an attempt to identify strategies to reduce maternal mortality.

## Discussion

This synthesis investigated how the 3DM has been used to categorise access barriers to obstetric care in LMICs, and if any changes were proposed over time. Five studies [6,9,42,44,45] applied this framework with its original categories. The other papers adapted the 3DM to account for context-specific features, which were not initially identified, but are relevant to understand if and how the journey to intrapartum care has changed over time. These alterations include highlighting new factors contributing to delays [7,29,30,32–37,40,41,43,46,47], proposing changes to the definition of delays to account for aspects previously overlooked [36–38], adding a fourth delay [15,29–31], and suggesting a change of perspective [14,15,28].

In the analysis of the First Delay, the adding of new contributing factors such as reliance on home birth, lack of birth preparedness and poor antenatal care attendance highlights an individual dimension which was not previously considered. In the Second delay, the attention to family and community actions to arrange transport, seemed to have neglected the existence of personal decisions. Although the woman’s status and her decision-making capacity were acknowledged in the First Delay; how her individual role contributes to (or prevents) each phase of delay has received little attention. Importantly, the studies in this synthesis, which have reported interviews with women, failed to investigate the journey to access care from the woman’s perspective. Furthermore, none of the studies explored the potential impact of women’s empowerment on the 3DM or the factors with the potential to achieve this. In fact, only four papers mentioned individual birth plans [30,35,40,43], of which only two [30,43] described any details in the results. None of these papers suggested using individual plans as a catalyst to mitigate the delays.

We also noticed that in all studies included the 3DM was applied retrospectively. All participants narrated their experiences after the birth and with a focus on the difficulties encountered. The sequence of events was observed when the delays had already happened and therefore a problem-identification approach guided the analysis (Figure 3). This has been pivotal to the documentation of household and health system’s challenges to accessing maternal care, but is less useful in identifying preventive interventions.

Clearly, the 3DM still has an important role in framing and documenting access barriers at every stage of the care-seeking journey. But its focus on barriers and a limited attention to the woman’s perspective seems to neglect the potential for an action-oriented approach. In this respect, the 3DM, with adaptation, could have greater utility by offering a framework for prospective identification of potential issues. This starts from understanding the woman’s position at family and society levels and acknowledging and valuing her individual health needs. Information on barriers should not be disregarded, but can be used as a foundation to build a positive childbirth experience centred on the woman. As also suggested in two of the studies included in this synthesis [14,15] a problem-solving approach seems to constitute the way forward.

**Figure 4.**
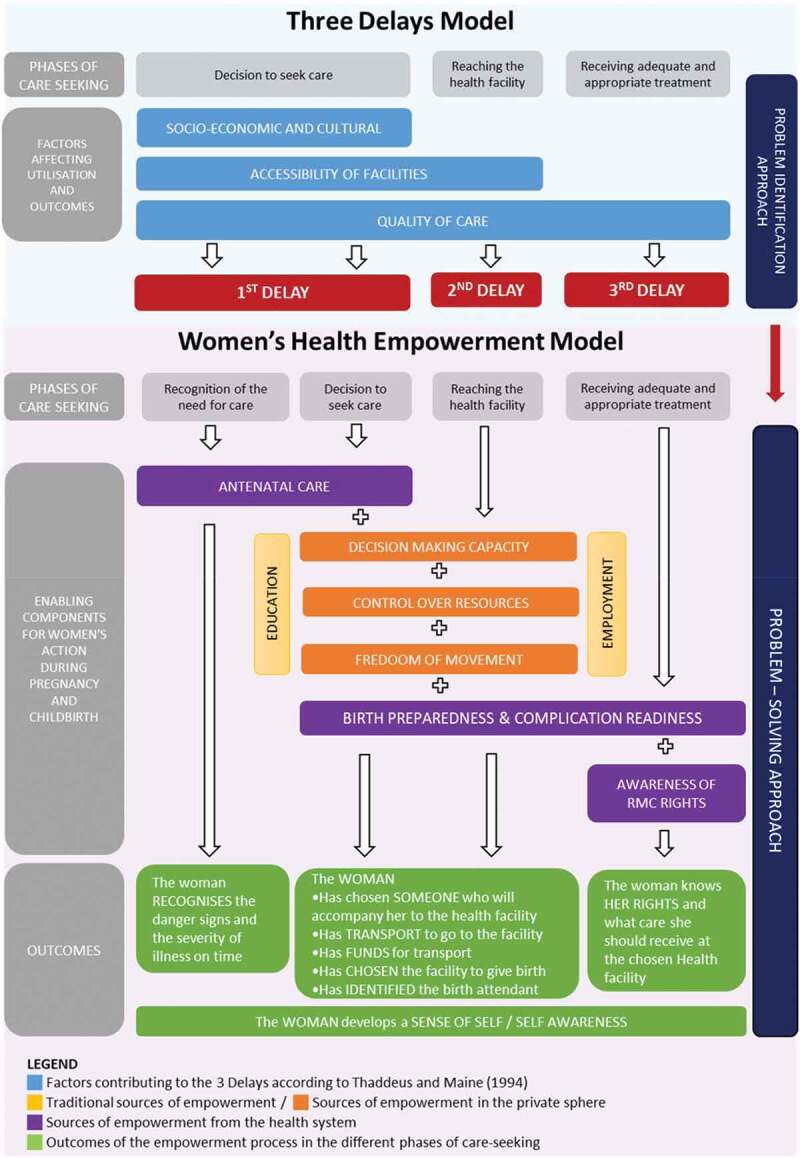
The women’s health empowerment model, developed by the authors.

To guide women (and their caregivers) in the formulation of their birth plans, preventing the occurrence of delays, a new framework: the Women’s Health Empowerment Model (WHEM) is proposed (Figure 4). WHEM resonates from the insights and recommendations from the included studies and the literature on women’s empowerment [19,50–54].

Its eight components include: education [28,39], employment [28], antenatal care [7,30,33,35,36,38,42,45], decision-making capacity [6,9,28,35,38], control over resources [28,42], freedom of movement [28], birth preparedness and complication readiness [15,29,30,42,43], and awareness of respectful maternity care rights [36]. These are now presented and discussed.

### Phases of care-seeking

In the WHEM the recognition of the need for care and the decision-making process are split in two separate, but associated phases. The first is influenced by the woman’s health education; the second is often determined by the power-relationship in the family.

### Recognising the need for care

As discussed in the results, the non-recognition of the need for care depends on multiple components including poor knowledge of danger signs of pregnancy, unclear perception of the severity of illness, socio-legal issues [4], and previous homebirth without complications [33,34]. Antenatal care (ANC) has the potential to be a source of empowerment if the woman is in control of her childbirth experience [55]. Regular ANC appointment attendance can have a positive impact on pregnancy outcomes [56], and contribute to reduce perinatal mortality [57]. Prenatal care can build women’s trust in the health system, providing the first healthcare contact during pregnancy, and creates an arena for screening and diagnosis, disease prevention and provision of health education [58]. The latter ensures that the woman receives adequate information about the physiological, medical and behavioural aspects related to pregnancy and childbirth, so that an individualised plan can be developed based on her needs and wishes. Knowledge and skills acquired during ANC should enable the woman (and her companion) to recognise the need for care and take action [35]. However, ANC may not be sufficient; as documented in this review, there were instances in which women attended ANC [15,41,43–45] and danger signs were recognised [38,40,46,47] but action was delayed because of the status of the woman and her limited decision-making capacity.

### Decision to seek care

The woman’s socio-economic status has often been measured through education and employment. These sources of empowerment enhance women’s opportunity to access the formal market and obtain personal income [59]. They also increase the likelihood of skilled birth attendance and of institutional delivery [20]. In this regard, encouraging formal education of girls to promote their employability represents a vital strategy to build their autonomy and economic independence [59]. It also contributes to global efforts towards the achievement of Sustainable Development Goal 5 advocating for economic empowerment of all women and girls [21].

In this review, educational level and employment were important indicators to describe the woman’s socio-economic status but did not provide sufficient information about the degree of control over personal life. Several papers included in this synthesis [6,9,14,15,28–30,32,37–39,44–47], analysed decision-making capacity on health-related matters to gain a better idea of women’s power in the family. Bloom et al. [52] and Gabrysch and Campbell [28] have also suggested to consider control over resources and freedom of movement as important dimensions to explore independency in the personal sphere. These 3 components are included in the WHEM due to their leverage in determining a woman’s role in the care-seeking journey.

Decision-making capacity reflects the woman’s ability to decide the course of action to achieve personal goals [60]. It involves the freedom to formulate a purposeful choice [19], the autonomy to decide without others’ control [61] and the ability to pursue the choice independently [62]. This aspect illuminates the woman’s possibility to have a say about health matters such as whether or not to attend ANC, choosing the birthing facility and the birth companion. In this review, women’s choice appeared to be rarely considered [15,29,31,46] as family members [6,9,14,15,28–30,32,37–39,41,42,44–47] were the main decision-makers.

Control over resources corresponds to the economic dimension of empowerment and can appear as woman’s contribution to the household budget, the ownership of financial accounts and the ability to use money independently [19,20,53]. Through an exploration of women’s financial power at household level, one can understand the woman’s capacity to spend money without having to ask permission. In this synthesis, the majority of studies concentrate on the availability of finance at household level. Three papers [14,30,46] acknowledged that women’s autonomy was influenced by husbands’ control of the family budget.

Freedom of movement refers to the individual’s liberty to travel independently, either alone or in a group. This aspect is worth considering since in certain communities women’s mobility is sanctioned by a male member of the family [32,63] or is restricted by social and cultural norms of seclusion aim to protect family honour [54,64]. Decision-making capacity, control over resources and freedom of movement determine who decides to seek care, how the facility will be reached and constitutes the basis for birth preparedness and complications readiness (BPCR).

### Birth preparedness and complication readiness (BPCR)

BPCR refers to making plans for a normal birth, and anticipating alternative actions in case of an obstetric emergency [65]. In LMICs, birth plans involve choosing a facility to give birth and a trained birth attendant, identifying transport, saving money to cover for travel costs and medical supplies, and having a blood donor in case of an obstetric emergency [65]. BPCR is a component of antenatal care but in the WHEM is highlighted as an independent element to recognise that a positive childbirth experience depends on the knowledge acquired to prepare for labour and birth, and on the practical arrangements made to access the health facility.

### Receiving adequate and appropriate treatment

As presented in the 3DM, care provision at the chosen facility relies on clinicians attitudes and competences, availability of medical supplies, and adequate management [4]. At the facility, women can also play a role if they are aware of the care they should receive. Recent evidence demonstrates that educating women about their health rights constitutes an opportunity for a better childbirth experience [66,67]. This can be achieved through maternity open days [67,68], group ANC [55,69] and community-based initiatives providing a platform whereby women share their experiences and learn from each other about how to prepare for childbirth.

### Outcomes

Expected outcomes of this process include woman’s choice of a birth place and birth attendant along with clear logistical arrangements to get to the facility in different circumstances (i.e. night, rainy season), also if obstetric emergencies occur. During pregnancy, women will also acquire specific knowledge to prepare for labour and childbirth according to their desires and needs. This has the potential for enhancing their sense of self and self-awareness [70], which represent the individual capacity to reflect on oneself and to alter behaviour accordingly [71]. The development of self-acceptance ensures that women gain control of their choices and decisions in their lives [50], and include the capacity to set goals which have a personal meaning and are oriented towards acquiring power [60]. Including these dimensions as an outcome of the WHEM, can help us better understand if and how the woman’s role can be modified and enhanced by the process of empowerment implemented during pregnancy and childbirth.

### Implications for practice

The WHEM constitutes a multidimensional tool to support women (and their caregivers) in planning for their childbirth. By accounting for women’s socio-economic status and their role in the family and society, this framework disentangles the different factors which influence women’s capacity to make decisions and take actions related to their health. This explains why decision-making capacity, control over resources and freedom of movement are incorporated. Yet these dimensions draw attention to the degree of freedom and autonomy that women have (or do not have) in planning for their childbirth experience [52,72], and can guide interventions accordingly. The inclusion of ANC and birth preparedness represents opportunities for health professionals to reflect on how these two components can be moved from a simple checklist to a customised plan re-discussed at every contact. This could inform future research to explore how this service can be customised according to women’s status and needs.

The WHEM adopts a prospective approach to understand individual situations and anticipate drawbacks during the childbearing period, thus counteracting the occurrence of delays. Rather than general recommendations, this framework should be used as basis for discussion with each woman, along the continuum of care, to develop tailor-made plans meeting specific needs and respecting the local context and culture.

The model embeds the recommendations of the most updated guidelines on antenatal [58] and intrapartum care [73] promoting a positive childbirth experience by respecting women’s choices, autonomy and decision-making capacity [58]. However, further research is needed to pilot this new framework in LMICs and to assess its usability to discuss and formulate individual birth plans with every woman and at every pregnancy.

### Strengths and limitations

A comprehensive search strategy, confirmed by two authors (VAD, CB), and the inclusion of studies with higher grading added rigour to the process, and reliability to the study findings. In terms of limitations, we assessed the use of the 3DM through published literature, therefore were reliant on the level of detail incorporated which was sometimes limited. Quality appraisal guaranteed that included papers offered substantial qualitative findings to inform the different factors contributing to the delays. Secondly, we did not include quantitative research as we intended to assess how the authors used the 3DM to analyse individual experiences and formulate new interpretations. Through in-depth interviews and FGDs participants could expand on each phase of delays and reveal aspects which may have not emerged through quantitative data, structured around the existing categories. Finally, the new model proposed – WHEM – is currently untested. Field-based research in LMICs is needed for assessing its utility and usability in clinical practice.

## Conclusions

This synthesis has demonstrated the need for an individual perspective to childbirth. This has led the reframing of the 3-Delay Model into a Women’s Health Empowerment Model to guide women and their caregivers in the formulation of their birth plans. The WHEM contextualises the status of the woman in her family and in the society and allows consideration of the challenges she is facing in preparing for childbirth. By bringing together all these elements, the new model provides an opportunity for health professionals to discuss and develop tailor-made plans with the potential to prevent delays and empower women during pregnancy and childbirth.

## Supplementary Material

Supplemental Material

## Paper context

The 3-Delays Model has guided the identification of access barriers to obstetric care in low and middle-income countries. It has been critiqued for limiting the prioritisation of strategies to reduce maternal mortality. This synthesis highlights the need for an individual perspective to childbirth, and proposes the Women’s’ Health Empowerment Model incorporating the 3 delays. This new tool should be used prospectively for early identification of potential issues and to empower women during pregnancy and childbirth.

## Appendix A. Summary of studies included

**Table A1.** Studies using the 3-delays model without any modification (N = 5).

					Factors contributing to the delays			
No	Author and (ref) Year of publication Study aim	Country	Type of study Sample population	Method of data collection Key informants	1^st^ Delay	2^nd^ Delay	3^rd^ Delay	Individual plans of the woman
1.	**Urassa et al.,1997** To identify operational factors contributing to maternal death	Tanzania Ilala district Dar es Salaam Urban	*Mixed-method* 117 Maternal deaths	Verbal autopsy, including interviews Relatives and health staff, senior administrators and health institutions	Women’s status (decision-making by mother-in-law, husband, nurse) Tradition (use of traditional healer first)	Distance Long transfer (travel time)	Poorly staffed facilities (limited number of nurses and doctors, lack of motivation) Poorly equipped facilities (blood, lack of drugs and theatre not always working) Inadequate management (poor decision, wrong management)	
2.	**Barnes-Josiah et al., 1998** To analyse the complex interplay between the personal decision of Haitian women and their families to seek care when faced with an obstetric emergency	Haiti	*National prospective study with qualitative component* 12 Maternal death	Verbal and social autopsies Creation of case histories Husband, mothers, friends, neighbours, TBAs, physician and clinic personnel	Indirect cost of seeking care Perceived quality of care (known lack of personnel at nearest hospital)	Distance Lack of means of transport Cost	Poorly staffed facilities (inability to perform a c/section) Inadequate management (inability to diagnose and treat acute conditions, being given wrong treatment and early discharge, inability/unwillingness to arrange referrals)	
3.	**D’Ambruoso et al., 2010** To extend the standard VA to gather and present information on experiences of emergency care-seeking, from the perspective of family members who experience some or all of the relevant event.	Burkina Faso (Ourgaye: remote rural setting) Indonesia (Serang and Pandelang: rural settings)	*Mixed-method study* 70 Maternal deaths (Burkina Faso) 104 Maternal deaths (Indonesia)	Verbal autopsy Structed questionnaires with open-ended questions Relatives of the deceased women	Illness factor (no knowledge danger signs) Women’s status (decision-making by family members) Perceived accessibility (transport/cost) Previous experience of care (dissatisfaction with midwives) Tradition: belief in magic and God not requiring medical intervention	Distance Lack of means of transport (use of non-motorised vehicles, limited a night) Cost for transport (loans)	Poorly staffed facilities (lack of staff) Poorly equipped facilities Inadequate management (delayed treatment, care contingent to payment)	
4.	**Khan and Pradhan, 2013** *Indirect use of the model To understand the missed opportunities to save maternal lives and explore social dimensions contributing to maternal mortality	India Five districts: Palamu, West Singhbhum, Giridih, Godda and Gumla	*Cross-sectional study with qualitative data collection* 833 Maternal deaths	Verbal autopsy Interviews with husbands and mothers in law	Illness factor (+ knowledge of danger signs; perceived severity of illness but no action) Socio-legal issues (unsafe abortion) Economic status (poverty) Tradition (use of local remedies to treat danger sign before formal care)	Distance Lack of means of transport (not always motorised) Cost of transport	Poorly staffed facility (lack of competences to identify complications, provision of wrong treatment) Inadequate management (care not available at the first facility needing further referral)	
5.	**Sharma et al.,2017** To investigate recognition, decision-making and care-seeking among facilities who experienced a maternal death, a reported postpartum haemorrhage; sequence of care-seeking actions; the role of husbands in these processes; and how perception of risk influence decision-making	Nigeria Jigawa state: 24 districts	*Qualitative study included in an RCT* 10 Maternal death 10 Women with PPH	Illness narrative and qualitative group interviews Women with PPH. For MD: husband, family members, neighbours, TBAs	Illness factor (no symptoms recognition by family members, different perception of severity of conditions based on previous experience) Women’s status (decision-making mainly by husband, mother-in-law, sister, traditional birth attendants) Perceived accessibility (cost) Previous experience with care (fear of health workers, absence of providers) Tradition (fatalism, use of spiritual care before formal care)	Lack of transportation		

**Table A2.** Studies using the three delays model but adding new factors explaining the delays (N = 10). New factors are underlined.

					Factors contributing to the delays			
No	Author and (ref) Year of publication Study aim	Country	Type of study Sample population	Method of data collection Key informants	1^st^ Delay	2^nd^ Delay	3^rd^ Delay	Individual plans of the woman
1.	**Essendi et al., 2011** *Indirect use of the model To investigate poor urban Kenyan men’s and women’s views on the factors that hinder the uptake of formal obstetric care services	Kenya Nairobi slum of Viwandani and Korogocho	*Qualitative study* Women with obstetric complication (sample size not reported)	Interviews and focus group discussion (16 in total) Women, male partners, Traditional Birth Attendants; Female opinion leaders; Male opinion leaders.	Illness factor (+ knowledge of danger sign)History of uncomplicated delivered limited cause for alarm Women’s status (decision-making by mother-in-law and husband or TBAs) Economic status (unaffordable care) Tradition (TBA’s choice as affordable) Perceived accessibility (cost of transport at night, insecurity at night) Satisfaction with service (unfriendly staff/procedures)	Distance (insecurity at night) Lack of means of transport at night Cost for referrals Poor road network	Poorly equipped health facilities (blood, drugs) Inadequate management (poor referrals) Need to show proof of ANC attendance to be admitted Care contingent to payment (additional amount for complications) and detention without bills been cleared Disrespectful care: poor attitudes, preference for male birth attendant, fear of ill treatments	
2.	**Jammeh et al.,2011** To provide a better understanding of the barriers to timely access to emergency obstetric care resulting in perinatal deaths and in survivors of severe obstetric complications	The Gambia Bansang hospital Central river region rural	*Qualitative study* 20 women surviving severe obstetric complications	Individual in-depth interviews Women, family members and TBAs	Illness factor (+ knowledge danger signs/perceived severity of illness) Women’s status (decision-making by mother-in-law and husband) Indirect cost of seeking care (transport and food at hospital) Tradition (use of local remedies to treat danger sign before formal care)	Distance (remote rural areas) and dependence from ferry service Poor road network (rainy season) Lack of means of transport Lack of ambulances for referrals	Poorly equipped facilities (blood)	Care seeking decisions and procedure Process of getting care
3.	**Kabali et al., 2011** To report the circumstances around the occurrence of complications that lead to death or near-miss, drawing on the testimonies of women who survived serious complication, and of families of women who passed away.	Democratic Republic of Congo Five District of Kinshasa city, urban	*Qualitative study* 208 Near-miss women 103 Maternal deaths	Semi-structured interviews and verbal autopsy Near-miss women For deceased women: husbands, mothers, step mothers, sister, sister in law, friends, neighbours	Illness factor (knowledge danger sign but neglecting them, severity of illness not threatening) Perceived accessibility (cost for C-section not affordable) Tradition (traditional remedies when formal care failed)		Poorly equipped facilities (blood, theatre not available) Inadequate management (students not doctors for inability to pay) Payment before treatment Poor attitude of staff (negligence)	
4.	**Echoka et al., 2014** To explore the barriers to EmOC services by women who experienced life-threatening obstetric complications or near miss	Kenya Malindi district, costal urban	*Qualitative study* 30 Near-miss women	In-depth interviews Women	Illness factor (fair knowledge of danger signs) Economic status (home delivery to contain cost)	Poor road infrastructure Lack of ambulance (need to organise own transport) Multiple referrals	Poorly staffed facilities (unavailability of doctors) Poorly equipped facilities Inadequate management (long queue before being treated)	Info collected on birth preparedness and place of birth Results: women made plans with TBAs and organise their own transport
5.	**Gebrehiwot et al., 2014** To explore health service providers’ perceptions of facilitators and barriers to the utilisation of institutional delivery	Ethiopia Tigray, two rural districts: Ganta-afeshum, Kilte-awlaelo	*Qualitative study* 12 Health extension workers 4 Midwives	In-depth interviews	Illness factor (+ knowledge of danger signs) Perceived accessibility (family’s reluctance to ask for transport to non-relative) Tradition: home delivery preferred/beliefs around going to health facility while pregnant	Distribution and location of facilities (mountain setting) Poor road network Poor network connection to call an ambulance	Poorly staffed facilities (lack of competences to detect complications and organise referrals) Poorly equipped facilities (equipment, electricity, water Relatives on allowed in the delivery room Negative attitude of providers	
6.	**Lori and Starke, 2012** To explore the cause and circumstances surrounding maternal mortality and severe morbidity	Liberia One rural county	*Secondary analysis of maternal death and near-miss audits with interviews* 120 Near-misses’ women 28 Maternal deaths	Interview with open-ended questions Women (NM) For deceased women: family members, community members and health workers	Illness factor (+ recognition of obstetric complications) Culture (hiding the problem/self-treatment, use traditional remedies before formal care) Women’s status (decision-making by family)	Distribution and location of facilities (remoteness) Lack of means of transport Non-motorised transport Cost for transport Lack of assistance at night (safety) Seeking care at multiple levels before tertiary	Poorly equipped facilities (delay receiving blood)	
7.	**Cham et al., 2005** To describe the socio-cultural and health service factors associated with maternal death to create an operative understanding of the concept of access	The Gambia Central and upper river division, rural	*Qualitative study* 42 Maternal deaths	Verbal autopsy and group interviews Family members, in-laws, husbands Health staff: midwives, ambulance drivers, generator operators, lab personnel, taxi drivers, ferry captain	Women’s status (following advice of older women about when to seek care) Economic status (cost of care) Perceived quality of care (fear of punishment from providers, maternal care only available when clinics opens, poor attitude of staff) Belief (progress of labour follows times linked to Muslim prayer) Underestimation of severity of complications due to previous uneventful home birth	Distance (travel time) Poor road network Lack of motorised vehicles Prolonged transportation Lack of ambulances/fuel Multiple referrals (Seeking care at inappropriate level)	Poorly staffed facilities (doctors, lack of competences) Poorly equipped facilities (blood, cost of supplies) Inadequate management (delay in providing care) Information from providers about where to give birth not clearly understood	
8.	**Mgawadere et al., 2017** To used the 3DM to analyse the delays associated with all maternal deaths occurred in one year.	Malawi Mangochi, semi-urban	*Mixed-method study* 151 Maternal deaths	RAMOS survey, including verbal autopsy Creation of case histories Household members, relatives, TBAs, medical personnel	Illness factor (lack of knowledge of obstetric complications) Women’s status (no financial empowerment if husband not available) Economic status (low family income) Perceived accessibility (distance) Previous experience with care (bad, poor quality) Tradition: visit TBA or traditional healer first Not wanting to stay in labour ward for long period Uneventful previous deliveries at home	Poor road network Distribution and location of facilities Distance (travel time) Cost for transport Slow means of transports	Poorly staff facilities (limited number, lack competences) Poorly equipped facilities (drugs, supplies, equipment, blood) Inadequate management (wrong assessment, wrong diagnosis, wrong treatment, lack of treatment guidelines) Inadequate referral system (transport not arriving for referral)	
9.	**Pagalday-Olivares et al., 2017** To assess the feasibility, in terms of potential of and requirements, of eHealth solutions to improve maternal healthcare in remote areas of Kpando, Ghana	Ghana Kpando: 6 Island and 5 peninsulas of Lake Volta, rural	*Mixed-methods study* Pregnant women (sample size not indicated) 15 Health workers 12 Maternal care stakeholders	Semi-structured interviews and focus group discussion Women, TBAs, men, Midwives, Representative of education services, national health insurance, representative of private facilities	Education (lack) Tradition (TBA as preferred choice of care)	Poor road network Distribution and location of facilities (geographical setting) Lack of means of transport Cost for transport	Poorly staffed facilities Poorly equipped facilities Inadequate management (inconsistent communication) Healthcare providers lack of knowledge about clients’ previous complications Poor referral system	
10.	**Sk et al., 2019** To identify facility and community level factors that contribute to maternal deaths.	India West Bengal, urban	*Mixed-methods study (use of primary and secondary data)* 317 Maternal deaths	Verbal autopsies (40) + hospital records Family members, neighbours and relatives	Illness factor (lack knowledge of danger sign of pregnancy and severity of illness) Education (illiteracy and ignorance) Tradition and beliefs No birth preparedness No antenatal care Previous uncomplicated pregnancies	Lack of means of transport Poor condition of roads Cost for transport and treatment (delay to mobilise funds, borrowing money) Mean of transport (not motorised) Multiple referrals	Poorly staffed facilities (lack medical staff) Poorly equipped facilities (blood and medical supplies) Inadequate management (delay in starting treatment)	Birth preparedness included in the assessment but not discussed in the results

**Table A3.** Studies proposing a change in the definition of delay (N = 3).

					Factors contributing to the delays			
No	Author and (ref) Year of publication Study aim	Country	Type of study Sample population	Method of data collection Key informants	1^st^ Delay	2^nd^ Delay	3^rd^ Delay	Changes to the definition of delays
1.	**Rodríguez Villamizar et al., 2011** To define critical points of change in the maternal care process of care, guide the decision-making around the issue and support the strengthening of policies for the provision of services with a view of achieving the MDG of improving maternal death.	Colombia Bucaramanga	*Retrospective descriptive study* 10 Maternal deaths	Epidemiologic reporting cards, clinical histories, field visits with interviews Family members and obstetricians	Illness factors (no knowledge of danger sign) Late access to ANC thus late learning of danger signs Women’s unawareness of their health rights	Poor referral between facilities (difficulties between lower and higher-level facilities)	Poorly staffed facilities (limited number of staff, lack of competencies) Poorly equipped facilities (blood) Inadequate management (incorrect diagnosis and treatment)	**First Delay separated in 2 phases** Recognition of danger sign of pregnancies Decision making process around seeking care and action
2.	**Jithesh and Ravindran, 2016** To explore social and health system factors contributing to the higher proportion of maternal deaths in the district.	India Wayanad, Kerala, less developed district (rural)	*Qualitative study* 14 maternal deaths	Social autopsy with In-depth interviews Household members, gynaecologists, PHC medical officers, junior public health nurses	Women’s status (concealing their status for fear of being blamed by family members) Economic status (poverty) Perceived accessibility (remoteness) Perceived quality of care (fear of C-section) Domestic violence Previous birth at home	Distribution and location of facilities (remote location) Poor road condition Difficult accessibility during rainy season Multiple referrals (facilities in opposite directions)	Poorly staff facilities (poor competencies and accountability, fear for decision taken) Poor equipped facilities (blood) Inadequate management (poor stabilization of women before referral, sub-standard care)	Delay’s definition to capture delays due to multiple referrals 1st delay: delay of > 1 h from the occurrence of complications during pregnancy or delivery to actually starting the journey to appropriate health facility 2^nd^ delay: any delay of > 1 h from the start of the journey (where complication occurred) to reaching a facility where the woman receives definitive treatment.
3.	**Chalet et al., 2017** To reaffirm and challenge assumptions around the drivers of the first two delays and discuss implications for policy makers and programme implementers working at the household, community and health system levels.	Ethiopia, India Indonesia Nigeria Tanzania Uganda Nepal	*Qualitative multi-country study* 45 Maternal deaths 84 Maternal ill cases (PPH only) Events narrative Ethiopia: 51 India: 32 Indonesia: 16 Nepal: 32 Nigeria: 40 Tanzania: 48 Uganda: 48	Reports, facility-based records, discussions, in-dept interviews and focus group discussions Women groups, villages leaders, community health workers	Illness factors (partial recognition of danger signs) Perceived severity of illness influences the choice of care (biomedical vs traditional) Women’s status (decision-making by older women and husband) Perceived quality of care (bypassing lower level facilities due to low quality) Tradition (traditional healers before formal care)	Lack of means of transport	Poorly staffed facilities Poorly equipped facilities (medications and supplies) Inadequate management (poor quality care) Long waiting time because of referrals	First Delay separated in 3 phases: Recognition of potentially life-threatening maternal and new-born illness Understanding of illness severity Decision making process around care seekingCombined the 3-Delays Model to Pathway to survival to better explore the drivers of the first two delays.

**Table A4.** Studies adding a fourth delay (N = 4).

					Factors determining the delays			
No	Author (ref) Year of publication Study aim	Country	Type of study Sample population	Method of data collection Key informants	1^st^ Delay	2^nd^ Delay	3^rd^ Delay	Definition of 4^th^ delay
1	**Combs Thorsen et al., 2012** To identify the socio-cultural and facility-based factors that contributed to maternal death	Malawi Lilongwe, 2 urban hospitals	*Qualitative study* 32 Maternal deaths	Case notes reviews (VA) and in-depth interviews Healthcare staff, family members, neighbour’s, traditional birth attendants	Illness factor (no recognition of danger signs and severity of illness by woman and husband) Perceived accessibility (remoteness) Tradition (preference for home delivery/TBA instead of institutional care)	Distance Lack of motorised vehicles in rural settings Lack of means of transport (at night)	Poorly staffed facility (lack of staff, lack of competences and communication skills) Poorly equipped facility (medicine and blood) Inadequate management (incorrect diagnosis, inadequate clinical work, lack of monitoring, attentiveness)	The patients conceal the HIV status and religion to the provider thus delaying treatment
2	**MacDonald et al., 2018** To explore determinants of maternal mortality from the perspectives of women of near-miss maternal experiences and community members and their solutions to reduce maternal mortality in the community	Haiti Rural communities close to the mountains	*Qualitative study (Participatory Action Research)* 5 near-miss women	Semi-structured interviews Focus group discussions Women, men community leaders and traditional birth attendants	Illness factor (lack of awareness, denial of danger signs, waiting too long to seek care) Socio-legal issues (hiding pregnancy to partner for fear of financial worries) Women’s status (reduced ability to make informed choices, decision-making by partner) Economic status Perceived accessibility (cost of care and transportation) Tradition (local medicine before formal care) Negligence from the woman/ignorance of young women	Distance (time) Cost for transport	Poorly staffed facility (lack of competences) Disrespectful care (feeling unwelcomed, humiliated by HCW, lack of respect toward accompanying TBA)	Delays from community accountability for maternal death due to Lack of a system to support transports Trusting traditional practice endangering women (pressure) and use precious resources
3	**Wallace et al., 2018** To gain insights into what influences people’s decision to seek care antenatally and during labour and birth.	Timor-Leste 4 municipalities: rural, peri-urban and urban settings	*Qualitative study* 17 women with various reproductive history	Semi-structured interviews (17) and focus group discussion (9) Women and men/partners	Women’s status (husband’s decision of place of birth and reliance on mother’s in law and TBA to resolve issues at home) Economic status Perceived quality of care (facility birth perceived good but not allowing to perform cultural blessing, fear of not having privacy and family support) Short labour Limited birth preparedness ANC provide confidence of healthy pregnancy not requiring institutional birth	Distance Lack of means of transport Cost for transport Poor road conditions Lack of ambulance/late arrival	Disrespectful care: staff ignoring cultural practices and perceptions	Delay from perceptions of respectful quality care Misgivings about staff Perceptions of hospital environment and policies*Individual plans of the woman**Minimal exploration of birth preparedness*
4	**Påfs et al., 2016** To explore care-seeking and experiences of maternity care among women who suffered a near-miss event in the early or late stage of pregnancy, and to identify potential barriers and health system limitations to maternal survival	Rwanda Kigali, 3 hospitals, urban setting	*Qualitative study* 47 women	Naturalistic inquiry using open ended questions Women	Illness factor (fail to recognise the need for care) Socio-legal issues (keep pregnancy secret if outside marriage, fear of stigmatisation) Economic status (no support from partner, lack of national insurance) Perceived accessibility (unaffordability of health services) Perceived quality care (fear of being mistreated if coming too early) Belief (late disclosure of pregnancy because of fear of witchcraft) Tradition (traditional medicine because of delayed payments)	Problems in referrals (lack of transports and constrains communication between facilities)	Poorly staffed facilities (lack of staff) Poorly equipped facilities Inadequate management (incorrect diagnosis and treatment, wrong referral, cost not covered by insurance, receiving hospital unprepared for to handle an emergency) Disrespectful care: Poor patient-provider interaction and communication Fourth delay Disrespectful care (distrust in health staff) Self- treatment	**Third Delays split in 2 parts**: Third delay: Delay in receiving care Fourth Delay: Delay from perception of respectful quality care Splitting the identification of factors affecting the delays by distinguish between f**ormal care seeking and informal care seeking**

**Table A5.** Studies proposing other changes (N = 3).

					Factors determining the delays			
No	Author (ref) Year of publication	Country	Type of study Sample population	Method of data collection Key informants	1^st^ Delay	2^nd^ Delay	3^rd^ Delay	Other changes
1	**Castro et al., 2000** To seek a comprehensive knowledge of the characteristics of maternal mortality in this setting, and identify factors that can be modified through concrete interventions	Mexico 3 states: Queretaro, San Luis Potosi, Guerrero urban	*Qualitative study* 145 maternal deaths	Verbal autopsy with open ended questions Relatives: mother, sister, acquaintance, husband	Illness factor (not actions from women despite presence of danger signs) Women’s status (required partner approval before seeking care) Perceived accessibility (cost for transport, service and medicine) Perceived quality of care (previous negative experience) Beliefs (women’s needs to ensure complications, interpretation of sign and symptoms through a non-medical paradigm addressed via local remedies) Domestic violence	Distance (remoteness, marginality) Lack of means of transport Prolonged transportation time Seeking care at more than 2 facilities	Poorly staffed facilities (lack of staff, limited competencies and training) Inadequate management (incorrect management, early withdraw medications) Long waiting despite serious complications	Classification of factors contributing to the delays into: Subjective Interactional StructuralThese are used to formulate solutions
**2**	**Gabrysch and Campbell, 2009** Explore the scope of determinants of skilled birth attendance, including preventive care seeking for delivery in LMICs.	Multiple countries	*Literature review* 2 reviews 80 original studies		Socio-cultural factors (maternal age, marital status, ethnicity, religion, traditional beliefs, family composition, mother’s education, women’s autonomy) Perceived need of care (information availability, health knowledge, pregnancy wanted, perceived quality of care, ANC use, previous facility delivery, birth order, complications) Economic accessibility (mother’s occupation, Husband’s occupation, ability to pay) Physical accessibility (region – urban/rural, distance, transport, roads) Perceived quality of care	Economic accessibility (mother’s occupation, Husband’s occupation, ability to pay) Physical accessibility (region – urban/rural, distance, transport, roads)	Quality of care (preventive or emergency)	Distinguish between quality of emergency care from quality of preventive care
**3**	**Sorensen et al., 2011** To analyse the main dynamics and conflicts in attending and providing good quality delivery care in a rural setting in Tanzania	Tanzania Kagera region, nortwest, rural	*Qualitative study* 31 mothers 32 relatives 19 healthcare providers	Semi-structured interviews and Questionnaire Women, relatives, TBAs	Women’s status (decision-making by husband) Perceived quality of care (perceived incapability of local facilities to manage birth complications, home birth preferred because of closeness to family support) Tradition (trust in TBA to be safe) Fail to follow healthcare providers’ advices due to ignorance and culture	Distance Lack of means of transport Cost for transport for referral Travel at night challenging (fear of thieves and wild animals)	Poorly equipped facilities (limited availability of supplies) Inadequate management (staff not available at night or long wait before arrival)	Suggest a new model (Actantial model) with 4 components: Subject, Aim, Helpers, Obstacles To facilitate the identification of responsible agents and strategies of action to improve access to EmOC.
