# Supplementary material for: Utility of the three-delays model and its potential for supporting a solution-based approach to accessing intrapartum care in low- and middle-income countries. A qualitative evidence synthesis
Source: Glob Health Action. 2020 Oct 12;13(1):1819052. doi: 10.1080/16549716.2020.1819052 (PMC7580724; doi:10.1080/16549716.2020.1819052)
Supplement: Supplemental Material [file ZGHA_A_1819052_SM6211.zip › Supplementary material - Quality appraisal scoring.pdf]

Reframing the Three Delays Model by focusing on women's health empowerment during pregnancy and childbirth in low- and middle-income countries. A qualitative evidence synthesis

QUALITY APPRAISAL OF INCLUDED ARTICLES

Based on **Hawker et al, 2002**

Scoring: Good = 4 - Fair = 3 - Poor = 2 - Very poor = 1

9-15	16-22	23-29	30-36
9	18	27	36
Very poor	Poor	Fair	Good

	Question	1<br>Did they provide a clear description of the study?	2<br>Was there a good background and clear statement of the aims of the research?	3<br>Is the method appropriate and clearly explained?	4<br>Was the sampling strategy appropriate to address the aims?	5<br>Was the description of the data analysis sufficiently rigourous?	6<br>Have ethical issues been addressed? Has the relationship between researchers and participants been adequately considered?	7<br>Is there a clear statement of the findings?	8<br>Are the findings of tthis study transfearable to a wider population?	9<br>How important are these findings to policy and practice?	
**N.**	**Title and Reference**	**Abstract and title**	**Introduction and aims**	**Method and data**	**Sampling**	**Data analysis**	**Ethics and bias**	**Results**	**Transferability and Generalizability**	**Implications and usefulness**	**Total**
1	Piecing Together the Maternal Death Puzzle through Narratives: The Three Delays Model Revisited (Combs Thorsen et al., 2012)	3	3	4	3	4	3	4	4	4	**32**
2	The fourth delay and community-driven solutions to reduce maternal mortality in rural Haiti: a community-based action research study (MacDonald et al., 2018)	4	3	4	3	4	3	4	4	3	**32**
3	The decision to seek care antenatally and during labour and birth – Who and what influences this in Timor-Leste? A qualitative project exploring the perceptions of Timorese women and men',(Wallace et al., 2018)	4	3	4	3	4	3	4	3	4	**32**
4	Still too far to walk: literature review of the determinants of delivery service use', (Gabrysch and Campbell, 2009)	4	3	4	3	4	3	4	3	3	**31**
5	Health workers' perceptions of facilitators of and barriers to institutional delivery in Tigray, Northern Ethiopia' (Gebrehiwot et al., 2014)	3	3	4	3	4	3	4	4	3	**31**
6	Illness recognition, decision-making, and care-seeking for maternal and newborn complications: a qualitative study in Jigawa State, Northern Nigeria', (Sharma et al., 2017)	4	3	4	4	3	3	4	3	3	**31**
7	User and provider perspectives on emergency obstetric care in a Tanzanian rural setting: a qualitative analysis of the three delays model in a field study', (Sorensen et al., 2011)	3	4	4	4	3	3	4	3	4	**32**
8	Beyond the numbers of maternal near-miss in Rwanda - a qualitative study on women's perspectives on access and experiences of care in early and late stage of pregnancy', (Pafs et al., 2016)	3	3	3	3	3	4	4	3	4	**30**
9	Factors associated with maternal mortality in Malawi: application of the three delays model (Mgawadere et al., 2017)	4	3	4	2	3	3	4	3	3	**29**

N.	Title and Reference	Abstract and title	Introduction and aims	Method and data	Sampling	Data analysis	Ethics and bias	Results	Transferability and Generalizability	Implications and usefulness	Total
10	Praying until death: revisiting three delays model to contextualize the socio-cultural factors associated with maternal deaths in a region with high prevalence of eclampsia in India (Sk et al, 2019)	4	3	4	3	3	2	4	4	2	29
11	Summary findings from a mixed methods study on identifying and responding to maternal and newborn illness in seven countries: implications for programs', (Charlet et al., 2017)	4	2	3	2	3	3	4	4	4	29
12	A lost cause? Extending verbal autopsy to investigate biomedical and socio-cultural causes of maternal death in Burkina Faso and Indonesia (D'Ambruoso et al., 2010)	4	3	4	3	2	3	3	3	3	28
13	A critical analysis of maternal morbidity and mortality in Liberia, West Africa (Lori et al, 2012)	4	4	3	2	3	4	3	2	3	28
14	Exploring the feasibility of eHealth solutions to decrease delays in maternal healthcare in remote communities of Ghana (Pagalday-Olivares et al., 2017)	4	3	3	2	3	3	3	3	3	27
15	A Study on Maternal Mortality in Mexico Through a Qualitative Approach (Castro et al, 2000)	3	3	4	2	3	3	4	2	2	26
16	Barriers to emergency obstetric care services in perinatal deaths in rural gambia: a qualitative in-depth interview study' (Jammeh et al., 2011)	3	3	3	2	2	2	4	3	3	25
17	Identifying factors associated with maternal deaths in Jharkhand, India: A verbal autopsy study', (Khan and Pradhan, 2013)	3	3	4	3	2	2	3	2	3	25
18	Barriers to emergency obstetric care services: accounts of survivors of life threatening obstetric complications in Malindi District, Kenya' (Echoka et al., 2014)	3	2	3	4	3	2	3	3	2	25
19	Social and health system factors contributing to maternal deaths in a less developed district of Kerala, India (Jithesh & Ravindran, 2016)	3	4	3	3	3	3	3	1	2	25
20	Benefits of combining methods to analyze the causes of maternal mortality, Bucaramanga, Colombia (Villamizar, Ruiz-Rodiguez et al, 2011)	3	3	3	1	2	1	4	3	4	24
23	'Operational factors affecting maternal mortality in Tanzania'(Urassa, Massawe, Lindmark, & Nystrom, 1997)	4	2	4	2	1	1	4	3	2	23
24	The "three delays" as a framework for examining maternal mortality in Haiti' (Barnes-Josiah et al., 1998)	3	3	3	2	2	1	3	3	3	23
25	Maternal mortality in the rural Gambia, a qualitative study on access to emergency obstetric care (Cham et al., 2005)	3	3	3	2	2	2	3	3	2	23
21	Barriers to formal emergency obstetric care services' utilization', (Essendi et al., 2011)	3	3	3	2	2	1	3	3	3	23

N.	Title and Reference	Abstract and title	Introduction and aims	Method and data	Sampling	Data analysis	Ethics and bias	Results	Transferability and Generalizability	Implications and usefulness	Total
22	Complications of childbirth and maternal deaths in Kinshasa hospitals: testimonies from women and their families, (Kabali et al., 2011)	3	2	3	2	2	3	3	3	2	23

STUDIES EXCLUDED

26	Experiences of survivors of maternal near miss in Mexico: a qualitative study based on the three delays model (Ranges-Flores et al., 2019)	3	3	3	2	2	1	3	2	1	20
27	Delay in seeking and reaching Emergency obstetric care in Eritrea (Gebrehiwet and morrow, 2007)	2	2	3	3	2	2	1	2	2	19
